# Risk factors of occupational skin diseases among traditional batik manufacturing workers in Yogyakarta, Indonesia

**DOI:** 10.1186/s13104-022-06105-0

**Published:** 2023-01-06

**Authors:** Sri Awalia Febriana, Erdina, Kusuma Dewi, Yohanes Ridora, Agustina Anggraeni, Niken Indrastuti, Fajar Waskito, Katharina Oginawati, Ikeu Tanziha, Cita Rosita Sigit Prakoeswa

**Affiliations:** 1 Department of Dermatology and Venereology, Faculty of Medicine, Public Health and Nursing Universitas Gadjah Mada, Yogyakarta, Indonesia; 2 Faculty of Medicine, Public Health and Nursing Universitas Gadjah Mada, Yogyakarta, Indonesia; 3 Faculty of Civil and Environmental Engineering, Institut Teknologi Bandung, Bandung, Indonesia; 4 Faculty of Human Ecology, IPB University, Bogor City, Indonesia; 5 Faculty of Medicine, Universitas Airlangga / Dr, Soetomo General Academic Hospital, Surabaya, Indonesia

**Keywords:** Batik manufacturing worker, OSD, OCD, Risk factors

## Abstract

**Objective:**

Batik is one of the Indonesian traditional arts made by decorating fabric using the resist dyeing technique. Currently, batik manufacturing serves as an important source of livelihood in Indonesia. However, the production process of traditional batik involves a range of chemicals and some repetitive physical movements, making batik production inseparable from physical and chemical hazards which increase the risk of developing occupational skin diseases (OSD). This study aimed to identify the risk factors related to OSD, including occupational contact dermatitis (OCD), among traditional batik manufacturing workers in Yogyakarta, Indonesia.

**Results:**

The study demonstrated that working duration (≥ 8 h daily) and type of work (wet process only) were statistically significant as risk factors for developing OSD. While for OCD, our results found that gender (male), history of atopy, and type of work (all and wet phase only) were risk factors for OCD development among traditional batik manufacturing workers.

## Introduction

Batik is one of the Indonesian traditional arts with its exceptional beauty exemplified in the decoration of fabric using the resist dyeing technique. As an ancient tradition, batik processing has been practiced for centuries in Indonesia [1, 2]. Currently, batik manufacturing serves as an important source of livelihood in Indonesia. There are more than 5.849 batik motifs from all regions in Indonesia. According to the Minister of Tourism and Creative Economy of Indonesia, batik industry can absorb a workforce of more than 200 thousand people in more than 47 thousand business units [3]. The export value of batik is reported reached USD 533 million in 2020 [4].

The traditional batik manufacturing process consists of 4 working steps: (1) fabric preparation; (2) wax application; (3) coloring/dyeing; (4) wax removal [5]. The process involves a range of chemicals along with some repetitive physical movements, making batik production inseparable from physical and chemical hazards which increase the risk of developing occupational skin diseases (OSD).

A high incidence of OSD in the traditional batik home industry was previously reported. Research by Kusbandono in 1996 showed that 28.13% of the workers in a study in Yogyakarta suffered from OSD [6]. Similar results were obtained by Soebono in 1995, with the prevalence of OSD reaching 26.9% [7]. Occupational contact dermatitis (OCD), which is defined as an inflammatory skin disorder caused by work-related exposure to irritants or contact allergens [8], is well-recognized as the most frequent type of OSD and accounts for 90% of all cases [9, 10]. This study aimed to describe the risk factors related to the emergence of OSD, including OCD, among traditional batik manufacturing workers in Yogyakarta, Indonesia.

## Main text

### Methods

The study was a cross sectional study conducted at traditional batik manufacturers in 5 districts in Yogyakarta Province including Bantul, Kulon Progo, Sleman, Gunung Kidul, and Yogyakarta City. A total of 222 workers at traditional batik manufacturers who work in the production process, aged 18–70 years old, were not pregnant and breastfeeding, voluntarily agreed to be involved in this study.

Interviews were carried out using the validated Indonesian translation of The Nordic Occupational Skin Questionnaire (NOSQ-2002/LONG) which had been used for previous similar studies and published elsewhere [11]. Some modifications were made to meet the situation of traditional batik manufacturers. Interviews and dermatological examinations were performed by dermatologists.

Patch testing was done in 23 subjects who were clinically diagnosed as OCD, and in 37 non-OCD subjects. European baseline series (Chemotechnique®), textile series (Chemotechnique®), and some additional allergens specific to the batik production process (paraffin, brown indigosol, soga, and indanthrene) were used in this patch testing. We included the additional allergens based on previous reports of their potential allergenic effects according to the Material Safety Data Sheets (MSDS), National Institute for Occupational Safety and Health Institute (NIOSH) website, and Pubchem. The patch were removed after 48 h, and the results were read after 72 and 96 h after application, according to the International Contact Dermatitis Research Group guidelines [12].

All statistical analyses were performed using SPSS 20.0 (IBM Corp., Armonk, NY). In order to identify the risk factors for OSD and OCD, bivariate analyses were performed. The factors with a *p-value* < 0.25 were considered for the multivariate logistic regression. *P-value* < 0.05 in the final model was considered significant.

### Results

#### Subjects characteristics

Majority of the subjects clinically diagnosed having OSD were male (53.4%), aged between 41–60 years (31.4%), had no history of atopy before (59.7%), worked more than 8 h per day (55.2%) mainly on the dry process (48.3%), had been worked as batik artisan more than 10 years (75.9%), and those who don’t always used PPE (74.1%). While most of the workers who were clinically diagnosed with OCD has following characteristics: male (82.6%), aged between 41 and 60 years (54.8%), had no history of atopy (60.9%), worked less than 8 h per day (52.2%) on wet process only (52.2%), had been worked as batik artisan for more than 10 years (65.2%), and used PPE irregularly (60.9%) (Table 1). This study categorized the type of work into 3 groups: (1) dry process (1^st^ and 2^nd^ steps); (2) wet process (3^rd^ and 4^th^ steps); and (3) all types of processes.

**Table 1:** Frequency, bivariate, and multivariate analysis of risk factors for OSD and OCD development among traditional batik workers in Yogyakarta

Variables	Frequency		Bivariate Model		Multivariate Model	
	Yes	No	Prevalence Ratio (PR; 95% CI)	P-value	Odd Ratio (OR; 95% CI)	P-value
Occupational Skin Disease (OSD)						
Gender				*0.00*	-	-
Male	31 (53.4%)	53 (32%)	2.41 (1.30–4.43)		1.88 (0.91–3.90)	0.09
Female	27 (46.6%)	111 (68%)	-		–	–
Age group				0.30	–	–
18–40 years	16 (27.6%)	72 (43.9%)	0.6 (0.15–1.99)		–	–
41–60 years	38 (31.4%)	82 (50%)	1.16 (0.34–3.9)		–	–
≥ 61 years	4 (6.9%)	10 (6.1%)	Ref		–	–
History of atopy				0.29	–	–
Yes	32 (40.3%)	48 (33.1%)	1.23 (0.85–1.77)		–	–
No	47 (59.7%)	97 (66.9%)	-		–	–
Working duration daily				*0.00*	–	–
≥ 8 h	32 (55.2%)	53 (32.3%)	2.58 (1.39–4.75)		2.35 (1.22–4.68)	*0.00*
< 8 h	26 (44.8%)	111 (67.7%)	-		–	*–*
Length of work experience as batik artisan				*0.04*	–	–
≥ 10 years	44 (75.9%)	91 (55.5%)	2.01 (1.01–3.61)		1.78 (0.73–4.2) –	0.20 –
< 10 years	14 (24.1%)	73 (44.5%)	-			
Type of work All processes	15 (25.9%)	23 (14%)	1.75 (0.84–3.7)	*0.04*	– 1.59 (0.67–4.5)	– 0.32
Wet process	15 (25.9%)	33 (20%) 108	2.51 (1.16–5.44)		3.12 (1.07–5.32)	*0.03*
Dry process	28 (48.3%)	(66%)	Ref		–	-
The use of PPE				0.4	–	-
Not always	43 (74.1%)	121 (74%)	0.77 (0.41–1.43)		–	–
Always	15 (25.9%)	43 (26%)	-		–	–
Occupational Contact Dermatitis (OCD)						
Gender				*0.00*	–	–
Male	19 (82.6%)	65 (32.7%)	9.79 (3.2–29.57)		6.22 (1.57–24.62)	*0.009*
Female	4 (17.4%)	134 (67.3%	-		–	*–*
Age group				0.77	–	*–*
18–40 years	10 (43.5%)	78 (39.2%)	0.77 (0.15–3.95)		–	*–*
41–60 years	11 (47.8%)	109 (54.8%)	0.61 (0.12–3.06)		–	-
≥ 61 years	2 (8.7%)	12 (6%)	Ref		–	–
History of atopi				*0.03*	–	–
Yes	14 (60.9%)	71 (37.2%)	1.68 (1.12–2.52)		5.10 (1.71–15.26)	*0.004*
No	9 (39.1%)	128 (64.3%)	-		–	*–*
Working duration daily				0.32	-	–
≥ 8 h	11 (47.8%)	74 (37.2%)	0.96 (0.27–3.44)		–	–
< 8 h	12 (52.2%)	125 (62.8%)	-		–	–
Length of work experience as batik artisan				0.65		–
≥ 10 years	15 (65.2%)	120 (60.3%)	1.23 (0.50–3.05)		–	-
< 10 years	8 (34.8%)	79 (39.7%)	-		–	–
Type of work				*0.00*	-	–
All processes	7 (30.4%)	31 (15.6%)	7.45 (2.05–27.05)		19.12 (3.47–86.48)	*0.011*
Wet process	12 (52.2%)	36 (18.1%)	11.0 (3.35–36.16)		29.09 (3.47–123.7)	*0.002*
Dry process	4 (39.6%)	132 (66.3%)	Ref		–	–
The use of PPE				0.31	–	-
Not always	14 (60.9%)	150 (75.4%)	0.73 (0.21–1.22)		–	–
Always	9 (39.1%)	49 (24.6%)	-		–	–

^*^Significant values are in italic

#### Risk factors for developing OSD and OCD

In the final model using multivariate analysis, daily working duration and type of work were statistically significant risk factors of OSD development. Workers who work ≥ 8 h (OR = 2.35; 95% CI = 1.22, 4.68) were more likely to develop OSD than those who work less than 8 h on a daily basis. Also, those who work only on wet processes (OR = 3.12; 95% CI = 1.07, 3.52) were more likely to develop OSD compared to those who work on other processes, i.e. dry and all processes.

Covariates such as gender, history of atopy, and type of work were statistically significant risk factors for OCD development, according to multivariate analysis. The male workers (OR = 6.22; 95% CI = 1.57, 24.62) were more likely to experience OCD compared to females. Consistently, the workers who have a history of atopy (OR = 5.10; 95% CI = 1.71, 15.26) were more likely to develop OCD than those who have no history. Type of work process engaged by the workers such as wet process (OR = 29.09; 95% CI = 3.47, 123.7) or all processes (OR = 19.12; 95% CI = 3.67, 86.48) was more likely to cause OCD than dry process (Table 1).

### Discussion

#### Gender

Male was considered as a significant risk factor for developing OCD (OR = 6.22; 95% CI = 1.57–24.62). Meding in 2000 reported a different result finding that more females were affected by work-related skin diseases, which are usually presented as hand eczema [13]. This different result might be caused by more male subjects being engaged in the wet process than females in this study. Almost half of the male subjects (48.81%) worked specifically on the wet process, while only 5.07% of total female subjects worked on the same process. The impact of handling wet work on the emergence of OSD and OCD has been well identified in most studies [14, 15].

#### Age

Age was not associated with the development of OSD and OCD in this study. Only 4 out of 14 elderly subjects were diagnosed having OSD and 2 of them with OCD. In contrast, a study by Soebono in 1995 in which most of the OSD occurred in elderly subjects concluded that age was a significant risk factor [7]. Moreover, Luebberding in 2013 assessed the skin barrier functions in female subjects ranging in age 18–80 years which demonstrated a decline in sebum production among the elder population and increased skin surface pH in post-menopause women. The stratum corneum hydration and transepidermal water loss (TEWL) were equal in both younger and older groups. These changes might affect the skin barrier function, thus increasing the risk of skin diseases in the elder population [16]. One of the possible explanations for this difference is that 10 out of 14 elderly subjects (≥ 61 years) worked only on the dry process. Working only on the dry process showed a lower rate of skin diseases.

#### History of atopy

History of atopy was not significantly related to the prevalence of OSD in this study, however, it was a significant factor to develop OCD. Atopy was also not a significant risk factor for OSD in the study by Kusbandono conducted in Yogyakarta in1996 [6]. In contrast, A study by Indriani conducted in Surakarta, Indonesia in 2010 among batik manufacturing workers found that history of atopy was a significant factor in developing irritant contact dermatitis (OR = 5.37; p = 0.001) [17].

Experimental studies had demonstrated that a past history of atopic dermatitis (AD) enhanced the susceptibility to irritants, and reflected a higher mean base TEWL [18, 19]. Atopy increases the tendency of dermal layers to develop an irritation process and also requires a longer time to be healed [20]. Individuals with a history of atopy constantly have a damaged epidermal barrier which would increase the TEWL and ease the penetration of irritants and microorganisms [21]. Whereas for allergic contact dermatitis, most studies suggest a decreasing tendency of contact sensitization in patients with a history of atopy because of Th1-cell down-regulation [22–24]. However, some recent studies show that contact sensitization to common allergens, such as nickel, cobalt, thimerosal, and fragrance mix, occurred in patients with AD at least as frequently as in the general population [21, 25]. Moreover, a more recent study found the opposite result, showing higher contact sensitization rates in atopic individuals (65.0%) compared to those in non-atopic (57.4%) [26]. A possible explanation might be due to an easier penetration of the allergens caused by the damaged barrier function in patients with AD [25].

#### Length of work experience as batik artisan and working duration

Length of work experience was not associated with the development of OSD and OCD. Working duration in this study was a significant risk factor for the emergence of OSD, but not for OCD. This could be due to a longer duration of exposure to hazards (chemical or mechanical), i.e. repetitive friction which increased the prevalence of callus among the workers who worked mainly on the dry process. Long-term and repetitive friction would lead to keratinization hyperactivity of the stratum corneum, thereby thickening and callus will likely occur.

A study conducted among clothing manufacturing employees in Beijing reported that average working hours per day accounted for a significant risk factor for developing OCD [27]. Basically, the development of OCD did not depend merely on the duration of work, since it should depend on the characteristic of exposure during the work, i.e. irritant or sensitizer.

#### Type of work

The wet process was found as a significant risk factor for developing OSD and OCD in this study while working on all types of processes apparently contributed to OCD development. Wet work had been reported as one of the most important causes of damage to skin barrier function [14]. Hydration would induce alterations in the horny layer, with stratum corneum expansion happening for as much as 3 up to fourfold. Additionally, a large amount of water becomes collected within the intercellular spaces and disturbed intercellular lipid structure. This hydration of stratum corneum may eventually facilitate the penetration of substances, including those with irritant or sensitizer characteristics, and consequently, ease the occurrence of occupational allergy contact dermatitis (OACD) and occupational irritant contact dermatitis (OICD) [15].

#### Work safety standards and the use of personal protective equipment

Personal protective equipment (PPE) was regularly used by 25.2% (n = 56) of workers in this study. Appropriate use of PPE is one of the important measures to protect workers from occupational hazards [28]. Statistical analysis in this study, however, showed that inadequate PPE use was not significantly related to the emergence of both OSD and OCD.

Based on direct observations in the workplace, this finding could be due to some damaged protective equipment that was used by the workers. However, different results were shown in Kusbandono’s study in 1996, which found the lack of PPE use was a risk factor for developing OSD in the 3^rd^ and 4^th^ stages of the batik production process [6]. Soebaryo had a similar result showing rejection of PPE use was a significant risk factor to develop OSD (OR: 2.88, CI: 1.33–6.25) [1].

We further found some conditions that possibly increase the risk of the chemicals affecting subjects’ health, for example, some work activities were done close to where the subjects handled raw materials for eating, thus the chemical splash might potentially contaminate their food. We also observed that certain dyeing materials were bought in a package with no labels, including having no safety label marked as bio-hazard and no mixing instructions to which the workers should refer while handling the high-risk materials.

### Limitations

This study had some limitations in terms of the scope and target population. The study was limited to only batik manufacturing workers in Yogyakarta Province without considering any other batik manufacturing conditions in other regions of the country. Nevertheless, the sample population was obtained from all districts in Yogyakarta, which was designated as the ‘Batik City’ in 2014 by the World Craft Council.

## Data Availability

The raw data is available upon reasonable request from the corresponding author.

## Abbreviations

OSD: Occupational Skin Disease
OCD: Occupational Contact Dermatitis
OACD: Occupational Allergic Contact Dermatitis
OICD: Occupational Irritant Contact Dermatitis
TEWL: Trans Epidermal Water Loss
PPE: Personal Protective Equipment
AD: Atopic Dermatitis
